# Transcription and DNA methylation signatures of paternal behavior in hippocampal dentate gyrus of prairie voles

**DOI:** 10.1038/s41598-023-37521-2

**Published:** 2023-07-07

**Authors:** Nicholas J. Waddell, Yan Liu, Javed M. Chitaman, Graham J. Kaplan, Zuoxin Wang, Jian Feng

**Affiliations:** 1 Department of Biological Science, Florida State University, Tallahassee, FL 32306 USA; 2 Department of Psychology, Florida State University, Tallahassee, FL 32306 USA; 3 Program in Neuroscience, Florida State University, Tallahassee, FL 32306 USA

**Keywords:** Epigenetics and behaviour, Sexual behaviour

## Abstract

In socially monogamous prairie voles (*Microtus ochrogaster*), parental behaviors not only occur in mothers and fathers, but also exist in some virgin males. In contrast, the other virgin males display aggressive behaviors towards conspecific pups. However, little is known about the molecular underpinnings of this behavioral dichotomy, such as gene expression changes and their regulatory mechanisms. To address this, we profiled the transcriptome and DNA methylome of hippocampal dentate gyrus of four prairie vole groups, namely attacker virgin males, parental virgin males, fathers, and mothers. While we found a concordant gene expression pattern between parental virgin males and fathers, the attacker virgin males have a more deviated transcriptome. Moreover, numerous DNA methylation changes were found in pair-wise comparisons among the four groups. We found some DNA methylation changes overlapping with transcription differences, across gene-bodies and promoter regions. Furthermore, the gene expression changes and methylome alterations are selectively enriched in certain biological pathways, such as Wnt signaling, which suggest a canonical transcription regulatory role of DNA methylation in paternal behavior. Therefore, our study presents an integrated view of prairie vole dentate gyrus transcriptome and epigenome that provides a DNA epigenetic based molecular insight of paternal behavior.

## Introduction

In mothers, gene expression changes in the brain underlie the physiological and behavioral adaptations of pup-rearing^1^. In addition, accumulating evidence suggests that neural gene expression changes are associated with sexual experience in fathers of biparental species, where both parents participate in pup-rearing, and paternal care contributes to pup development^2^. However, the regulatory mechanism of gene expression underlying these processes remains elusive.

The prairie vole, *Microtus ochrogaster*, has become a valuable organism to model social bonding, where vole individuals mate exclusively, share nests, and exhibit biparental care of newborn pups^3–5^. Both mother and father voles nearly equally participate in parental behaviors (“Mother” and “Father” thereafter, respectively), such as pup grooming, huddling, retrieving, and nest building^6^. Though these parental behaviors normally present after a litter is born, they may spontaneously occur in some sexually naïve males when exposed to conspecific pups (“Parental”)^7,8^. While about 60% virgin males are parental, the other virgin males display aggression towards conspecific pups (“Attacker”)^9–11^. Therefore, studying the behavioral dichotomy in male prairie voles may contribute to our understanding of paternal care.

Environmental stimuli play a large role in parental behavior manifestation and modulation during post-partum periods, which also affect gene expression through epigenetic modulations^12,13^. For example, it was demonstrated that parental behaviors in response to pup exposure can be altered by using compounds affecting epigenetic states, such as histone deacetylase inhibitors^14^. Furthermore, differential DNA methylation has been observed in the hippocampus of rodent offspring upon altered maternal care^15^. Although DNA methylation, a major epigenetic mechanism, has been implicated in various basic brain functions and diseases^16–18^, the potential role of DNA methylation in paternal behaviors remains largely unknown.

In prairie voles, dentate gyrus (DG) of hippocampus expresses receptors for oxytocin^19^, a key molecule in pair bonding and parental behaviors. It was found that oxytocin receptors are subjected to DNA methylation mediated gene expression regulation^20^, where the density of oxytocin receptors in DG is associated with mating tactics and reproductive success in male voles^21^. Furthermore, mating and social interaction, which lead to pair bond formation, have been found to modulate neural precursor cell proliferation and differentiation in the DG of parental voles^22,23^. While pup exposure elicited cell proliferation^24^, fatherhood decreased cell survival in the DG^25^. In addition, exposure to psychostimulant drugs, such as amphetamine, not only diminished pair bonding^26,27^, but also impaired social recognition and decreased neuronal and neurochemical activation in the DG^28^. Although the evidence is sporadic, taken together it suggests a role of DNA methylation in DG’s function in prairie vole social behaviors. However, it remains unknown how gene expression and DNA methylation changes occur at the genomic scale and their potential interplay in parental behaviors. To address this question, we examined the prairie vole DG transcriptome and DNA methylome aiming to explore molecular insights of parental behaviors, particularly the paternal behavioral dichotomy in virgin males.

## Materials and methods

### Animal Subjects

Subjects were sexually-naïve male and female prairie voles, *Microtus ochrogaster*, from a laboratory breeding colony. Subjects were weaned at 21 days of age and housed in same-sex sibling pairs in plastic cages (12 W × 28 L × 16 H cm) containing cedar chip bedding with water and food provided ad libitum. All cages were maintained under a 14:10 light:dark cycle, and the temperature was kept at 20 °C. Adult subjects (at 90–120 days of age) were randomly assigned into experimental groups, where voles destined to be pair-bonded were paired and cohoused, and sexually naïve males were continuously housed in same-sex sibling pairs. Females gave birth following 21–23 days of pairing with a male, and the mother and father voles were continuously housed with their offspring. At three days postpartum, mothers and fathers (i.e. “Mother” and “Father” groups, respectively) were tested for their parental behaviors towards a conspecific pup. Age-matched virgin males were also tested for their spontaneous parental behaviors towards a conspecific pup. Depending on whether virgin males displayed parental behaviors towards pups or attacked pups^10,29,30^, they were classified into “Parental” and “Attacker” groups, respectively. All animal experimental procedures were approved by the Florida State University Institutional Animal Care and Use Committee and were in accordance with the U.S. National Institutes of Health Guide for the Care and Use of Laboratory Animals^31^. The study was reported in accordance with ARRIVE guidelines.

### Parental Behavior Test

The parental behavior test was conducted as previously described^10,29,30^. Briefly, all subjects were tested in a plexiglas cage (20 W × 45 L × 25 H cm) with a thin layer of cedar chip bedding and ad lib food and water, as described for the housing cages. The subject was placed in the testing cage and allowed for a 15-min habituation. Afterwards, an unfamiliar stimulus pup (at 3-day age) was introduced into the testing cage at the opposite corner from the subject, and the subject’s behaviors were digitally recorded for 60 min. The “Attacker” virgin males were immediately separated from the pup once they showed aggression with latency of the first attack scored. The “Parental” virgin males, “Father”, and “Mother” groups’ behaviors were recorded and scored by a trained experimenter blind to the treatment groups using JWatcher software program v1.0 (https://www.jwatcher.ucla.edu). The duration and frequency of the subject’s interactions with the stimulus pup within the first 10-min were quantified. The scored behaviors included both parental behaviors (pup huddling, pup carrying, licking and grooming, and nest building) and non-parental behaviors (auto-grooming, locomotion, olfaction, and resting)^8,29,32^.

### Behavior data analysis

Group-wise differences in all behavioral measurements were analyzed using a one-way ANOVA. Post-hoc analyses were conducted using Tukey’s HSD tests (p < 0.05). Plots were generated from GraphPad v9.3.1.

### Brain tissue collection

After the parental behavioral test, subjects were immediately decapitated without anesthesia. Brains were extracted and immediately frozen on dry ice. Brains were sliced into 200 µm sections on a cryostat and thaw mounted on slides. Thereafter, 1 mm-diameter punches from 4 consecutive sections were taken bilaterally from the DG of the hippocampus^33^. Tissue punches were stored at -80ºC until further processing.

### Next generation sequencing library preparation

DNA and RNA were isolated from the same tissue using the Qiagen AllPrep DNA/RNA Micro Kit (Qiagen, #80284) according to the manufacturer’s protocol, including treatment of RNA with RNase-free DNase Set (Qiagen, #79254), and then quantified by Qubit fluorometry. The RNA integrity (RIN) was determined using the Tapestation 4200 system (Agilent, # G2991BA), and samples with RIN ≥ 8 were chosen for downstream applications. Each sequencing library was prepared from DNA or RNA isolated from a single vole brain. Total 23 RNAseq libraries (6 Attacker, 6 Parental, 5 Father, 6 Mother) and 23 reduced-representation bisulfite sequencing libraries (6 Attacker, 6 Parental, 5 Father, 6 Mother) were included in the study.

For each sample, 150 ng of total RNA from a single animal was applied to the NEBNext rRNA Depletion Kit (New England Biolabs, #E6310L) according to the manufacturer’s protocol. Ribo-depleted RNA sequencing (RNA-seq) libraries were constructed using the NEBNext Ultra II Directional RNA Library Prep Kit for Illumina (New England Biolabs, #E7765S) according to the manufacturer’s protocol. Following RNA fragmentation and cDNA conversion, ends of cDNA fragments were ligated with universal Illumina adapter sequences. RNAseq libraries were individually indexed with NEBNext Multiplex Oligos for Illumina (New England Biolabs, #E7335S) and amplified for 11 cycles of PCR amplification. All clean-up steps were accomplished using the supplied purification beads within the NEBNext Ultra II Directional RNA Library Prep Kit. RNA-seq libraries were then sequenced 50-bp paired-ended on an Illumina NovaSeq 6000 Sequencer with a 5% PhiX spike-in control.

Reduced representation bisulfite sequencing (RRBS)^34^ libraries were prepared using a Premium RRBS Kit (Diagenode, #C02030032) according to the manufacturer’s protocol. Briefly, 100 ng high-quality genomic DNA from one single animal was digested with MspI, end-repaired, ligated to adapters, and then size selected using AMPure XP beads (Beckman Coulter, #A63881). All size-selected samples were treated with sodium bisulfite conversion. Spike-in control DNA was included for the monitoring of bisulfite conversion efficiency. Libraries were then purified after 14 cycles of PCR amplification. An Agilent Bioanalyzer and KAPA Library Quantification Kit were run to assess library quality and quantity. RRBS libraries were sequenced 100-bp single-ended on an Illumina NovaSeq 6000 Sequencer with a 5% PhiX spike-in control.

### Quantitative PCR and analysis

400 ng of DNase-treated RNA was converted to cDNA using qScript cDNA SuperMix (Quantabio, #95048). Quantitative PCR (qPCR) was performed using cDNA, specific primers for each gene of interest (see below for primer sequences), and PerfeCTa SYBR Green SuperMix Low ROX (Quantabio, #95056) on a QuantStudio 7 Flex Real-Time PCR 384-Well System (Applied Biosystems, # 4485701). Reactions were set up in a total volume of 10 μL containing 5 μL of SuperMix, 0.5 μL each of forward and reverse primers (10 μM), 2 μL of diluted cDNA template (4 ng/μL) and 2 μL of nuclease-free water, and run according to the manufacturer’s standard cycling protocol: 2 min at 50 °C, 3 min at 95 °C; 15 s at 95 °C, 45 s at 60 oC (40 cycles); followed by a default dissociation program to assess the specificity of PCR products. The primer pairs were tested for > 90% efficiency by standard curve and the reactions were confirmed to have a single melting curve peaked at the right temperature, before further analysis. For each gene target, 7–8 samples per condition were run in duplicate.

The threshold cycle (Ct) values were obtained using the QuantStudio Real-Time PCR Software v1.2 (Applied Biosystems). Relative gene expression levels were calculated using the 2^(-−ΔΔCt)^ method with *Sdha,* which is stably expressed in the ventral hippocampus^35^, as the internal reference gene for data normalization.

Group outliers were identified using the Graphpad Outlier webtool (https://www.graphpad.com/quickcalcs/grubbs1/) and removed from final analyses, resulting in 5–8 replicates per condition for each analysis. The results are presented as fold change relative to the ‘Father’ group. Statistical analyses were performed using Student's t-tests, where the assumption of equal variance was tested using the F-test. Figures were generated using GraphPad v9.3.1.

Primer Sequences:

Chrna3 FGGAAGCCCTCTGACTACCAA.

Chrna3 RAAAGATGGCTGGAGGGATCC.

Chrnb4 FTCCCAGCTCATCAGTGTGAA.

Chrnb4 RAGGTCCCATCGGCATTGTTA.

Col12a1 FAGTGTGCCGGTTATAGGTCA.

Col12a1 RAAAGCAGACACAAGAGCAGC.

Crhr2 FAACACGACCTTGGACCAGAT.

Crhr2 RTTGACAATGAGGGCGATTCG.

Fzd10 FTTCTTCCTGTGCTCGCTGTA.

Fzd10 RCAGGTAGTTGGGGTCGTTCT.

Fzd7 FGGGCTGCTATTTCATGGTGG.

Fzd7 RGGAACCAAGTGAGGGACAGA.

Igf2 FCTCATCTCTTTGGCCTTCGC.

Igf2 RCAACACTCTTCCACGATGCC.

Kiss1r FGGTTCCCTTGTTCTTCGCTG.

Kiss1r RGACGCAGCACAGTAGGAAAG.

Oprm1 FATTCACCCTCTGCACCATGA.

Oprm1 RGAGAACGTGAGGGTGCAATC.

Prlhr FCATCCTCCTGTCCTACGTCC.

Prlhr RTCCCGAAGCAGGTTGAAGAT.

Sdha FAAGAGGACAACTGGAGGTGG.

Sdha RTGAGGCTCTGTCCACCAAAT.

Tcf7l2 FACAGCAACGAACACTTCACC.

Tcf7l2 RCCTCCTGTCGTGATTGGGTA.

Wnt6 FCCCTGGTCATGGATCCTACC.

Wnt6 RGGCTGTCTCTCGAATGTCCT.

### Sequencing data pre-processing

Raw sequencing reads were first evaluated for quality using FastQC v0.11.9 (https://www.bioinformatics.babraham.ac.uk/projects/fastqc/). In our RNAseq analysis, we produced a range of read pairs between 17 and 58 million. Meanwhile, in the reduced-representation bisulfite sequencing, we generated a span of single-ended reads ranging from 30 to 49 million. To address a positional sequencing error within the RNAseq library, where an entire tile within the sequencing chip had extremely low quality, FilterByTile^36^ was applied without incurring biases on the rest of the dataset. Afterwards, sequencing reads were trimmed of adapters and to a minimum of 20 quality score and 20 read length using TrimGalore v0.6.4 (https://www.bioinformatics.babraham.ac.uk/projects/trim_galore/). RRBS sequencing reads were trimmed with TrimGalore’s RRBS mode which eliminates synthetic cytosine signals from the ends of reads that was incorporated during the end-repair process. Reads were checked again for quality after trimming, before further analysis.

### RNAseq analysis

#### Alignment, assignment, and differential expression analysis

Alignment of RNAseq reads was performed using the splice-junction aware alignment software STAR v2.5.4b^37^ with the MicOch1.0^38^ annotation set as the reference genome. Aligned reads were assigned and counted to gene-level features using FeatureCounts v2.0.0^39^.

Gene-level counts from RNAseq reads were imported to R and analyzed using EdgeR v3.28.1^40^. Genes with low counts and those missing counts from at least half of the samples per group were removed from the analysis, resulting in about 70% of annotated genes to be considered in downstream analyses. To facilitate experimental group representation, we utilized the “plot_MDS” function to calculate coordinates for each sample in Euclidean space. The coordinates were visualized using ggplots2 v3.3.3^43^, and. We used the “stat_ellipse” function with a level parameter set to 0.8. We performed a robust Principal Component Analysis (PCA) implemented in the rrcov R package^41^ and identified one RNAseq sample in the Father group with increased orthogonal distance from the rest of the samples in the study, which met criteria for outlier and was excluded from further analysis^42^. Dispersion, biological coefficients of variation (BCV) and normalization factors for the dataset were subsequently estimated. RNAseq samples were evaluated in two-dimensional space using multi-dimensional scaling to determine whether certain principal components are driving the variation among and between groups. Then, the RNAseq design matrix and generalized linear models were created contrasting the four groups of voles (“Attacker”, “Parental”, “Father”, and “Mother”) in the experiment. The “Mother” group was only compared to the “Father” group, as both have experienced pair-bonding, while the virgin male vole groups (i.e., “Attacker” and “Parental”) have not, to reduce confounding variations in the analysis. Hypothesis testing was performed through the likelihood ratio test and any genes with a log_2_ fold change less than − 0.5 or greater than 0.5, and p value < 0.05 were claimed to be a differentially expressed gene (DEGs). These results were represented using volcano plots generated using ggplots2 v3.3.3^43^.

#### Gene ontology enrichment analysis

Differentially expressed prairie vole genes were annotated to the mouse orthologous gene IDs using Ensembl’s biomart^38^ annotation database and SQL manipulations, before they were applied for Kyoto Encylcopedia of Gene and Genomes (KEGG)^44^ pathway analysis, which overcomes KEGG’s lack of annotation for prairie voles. The analysis was performed using a web-based tool, WebGestalt^45^, a hypergeometric overlaps test. Significant pathways were considered by an FDR value < 0.05. For biological process gene ontology pathway enrichment testing, prairie vole gene names were passed to gProfiler^46^ for gene ontology over-representation analysis using Ensembl’s^38^ prairie vole annotation. Enrichment ratio is defined by the following formula:

$$\frac{Intersection\,Size}{\left(\frac{Term\,Size}{Effective\,Domain\,Size}\right)*Query\,Size}$$

#### Rank–rank hypergeometric overlaps

Rank–rank hypergeometric overlaps (RRHO) analysis identifies overlapping transcriptome expression profiles without pre-set thresholds, and determines the degree and the direction of overlapping genes^47^. An improved version of RRHO is applied to allow discordant signatures to be assessed as robustly as concordant signatures. With this, visualization of each quadrant is separated, where the lengths of each side representing the relative length of each input gene list^48,49^. Each expression list was ranked by multiplying the − log_10_(p value) and the sign of the log_2_ fold expression change. RRHO difference maps were generated by representing the − log_10_, Benjamini and Yekutieli adjusted p value from the hypergeometric test^50^.

#### Differential expression clustering

All genes that had a significant expression change between experimental groups (i.e., “Attacker” vs “Parental”, “Attacker” vs “Father”, “Parental” vs “Father”, and “Father” vs “Mother”; Table S2) were collected, and normalized gene counts were formatted into a table in R. Z-scores were calculated for each gene and passed to the pheatmap v1.0.12^51^ package for hierarchical clustering using k-means clustering based on Euclidean distance. All eight clusters were named in roman numbers I–VIII.

#### Gene ontology clustering

Genes in cluster VI of the “Differential Expression Clustering” analysis were taken for evaluation of over-represented ontologies. To simplify the interpretation, the gene ontology pathways were further divided into 10 clusters (named in Arabic numbers 1–10), and a network was constructed using Gene Ontology Markov Clustering (GOMCL)^52^. The resulting network and annotation table were passed to Cytoscape v3.9.1^53^ for network visualization.

### RRBS analysis

#### Alignment, methylation calling, and differential methylation analysis

Bismark v22.3^54^ genome preparation tool was used to create appropriate reference genomes for bisulfite sequencing alignment. Subsequently, quality-checked RRBS sequencing reads were aligned using the bowtie2 based methylation alignment algorithm in the Bismark suite with increased seed extension effort (parameters: -N 1, -L 20, -D 20). To extract methylation status from the alignment data, a methylation extractor tool of Bismark is applied to create a sample-wise list of CpG positions with the number of reads at that location with methylated calls and unmethylated calls. The resulting files were formatted and imported to R for differential methylation analysis. Differential methylation was calculated using a similar design structure to the differential expression analysis, which was processed using DSS general v2.34.0^55^ that implements a bayesian hierarchical model for dispersion estimation of the beta binomial distribution. Differentially methylated CpG sites (DMCs) were those from the Wald testing procedure with p value thresholding < 0.05 and an absolute methylation difference of 15%. The data was represented by a volcano plot created using ggplot2^43^. Differentially methylated regions (DMRs) were calculated using DSS general v2.34.0^55^, using default parameters for length and number of CpGs required per region.

#### Gene annotation, KEGG pathway, and genomic feature distribution analysis

DMCs were annotated to an imported prairie vole genome using a Homer suite tool^56^, where those located in gene promoters and gene bodies were assigned to the corresponding genes. Gene promoters were defined as the region from 2000 base pairs upstream to the transcription starting site (TSS) of each gene. Gene bodies referred to the region from TSS to 1000 bp downstream of transcription termination site (TTS). The associated annotated gene names, were evaluated for over-represented KEGG pathways, as mentioned above, using the orthologous mouse gene annotation, GRCm38^38^ from Ensembl’s Biomart database.

To evaluate the genomic feature distributions between the differentially methylated CpG dataset and a set of CpG sites that were possible to capture through our sequencing method, we in-silico digested the reference genome MicOch1.0 for prairie voles using the MspI enzyme, which cuts at C^CGG regions. After collecting 100 bp regions in the 3′ and 5′ direction of each restriction site and counting the number of CpG dinucleotide regions from the fragment sequences, we annotated the fragments to the reference annotation in the same manner as listed above for the DMCs. Once we had a reference set and the differentially methylated CpG genomic feature distributions, we performed hypergeometric tests to see whether a specific category of genomic feature was under- or over-represented in the differential analysis with Benjamini–Hochberg corrected p values^50^. These analyses were performed using in-house R code and python scripts.

### RNAseq and RRBS correlation analysis

Differentially expressed genes with DMCs were chosen for RNAseq and RRBS correlation analysis. The analysis only included the differentially methylated sites located at gene promoters and gene bodies, and their correlation with transcription changes were done separately. First, for all differentially expressed genes that have DNA methylation changes within the gene body, they were separated into eight quantiles (i.e., 12.5% consecutive increments) according to the value of log_2_ fold gene expression change. Furthermore, for all differentially expressed genes that have differentially methylated sites in their promoters, they were divided into four quartiles (i.e., 25% consecutive increments) based on the value of log2 differential gene expression fold changes. Finally, for either gene body or gene promoter analysis, spearman’s correlation was applied to examine any significant correlation between transcription and DNA methylation changes within each of the 8 quantiles or 4 quartiles, respectively. When surveying DEGs with DMCs found in their promoter, we reduced the number of quantiles because there were less observations available. These correlations were corrected for multiple testing through significance thresholding by Bonferroni correction^57^.

Furthermore, we evaluated the over-represented biological pathways on all genes that had both transcription change and differential DNA methylation in promoter or gene body regions. The analysis was done in each of the four comparisons separately (i.e., “Attacker” vs “Parental”, “Attacker” vs “Father”, “Parental” vs “Father”, and “Father” vs “Mother”), but without segmenting the differential expression data into quantiles. They were evaluated for over-represented KEGG pathways using the annotated mouse gene IDs as mentioned above. These pathways were imported into Cytoscape and were used to construct a similarity network within the EnrichmentMap^58^ plugin. The ClusterMaker^59^ plugin was used to cluster the network using the affinity propagation algorithm^60^ by identifying “exemplars” or highly connected nodes. The resulting clustered network was visualized using Cytoscape v3.9.1^53^. Cluster labels were assigned using the AutoAnnotate^61^ plugin which takes network node information and automatically assigns cluster labels according to a word tag cloud.

## Results

### Behavior

**Figure 1:**
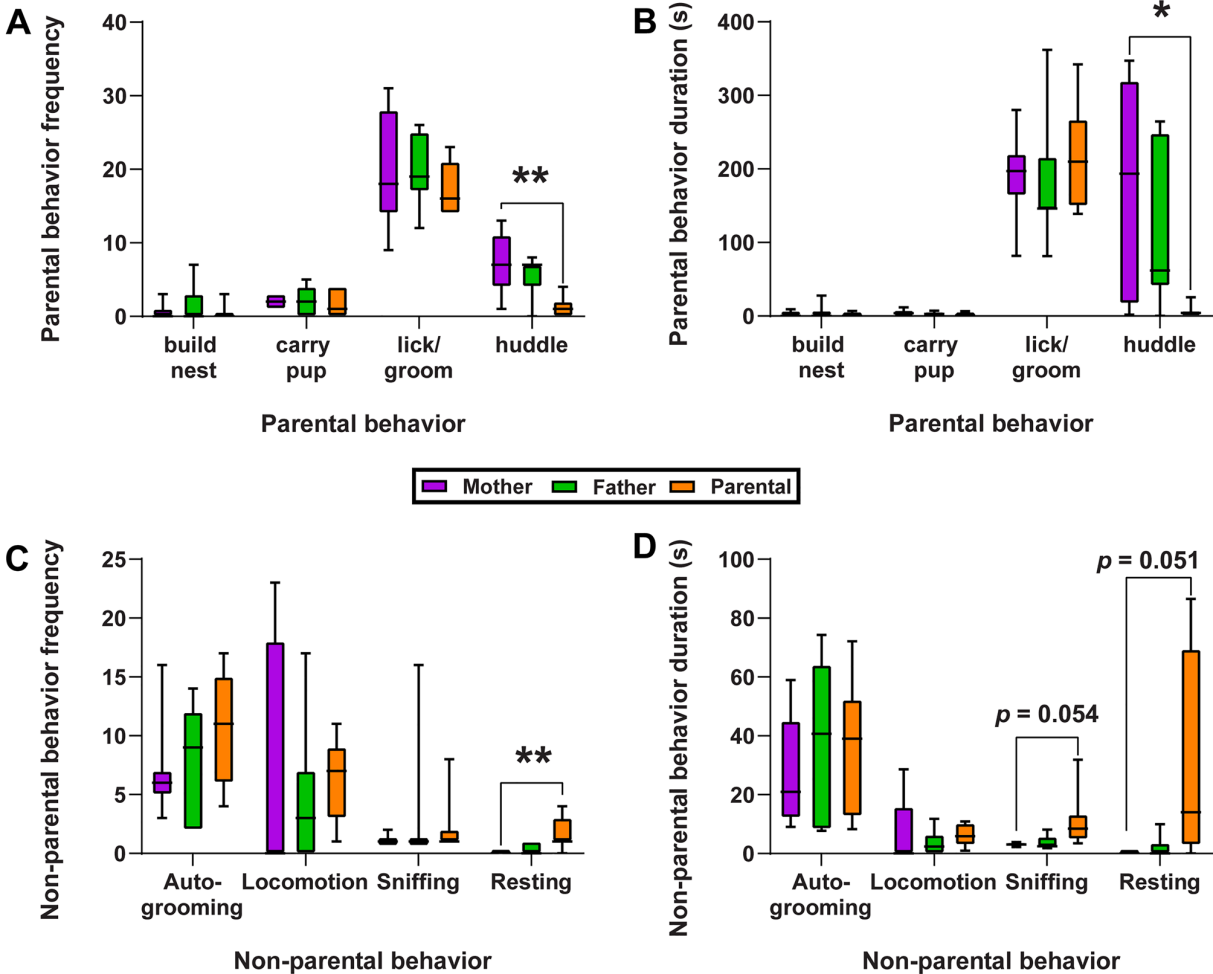
A subset of sexually-naïve male voles exhibit spontaneous parental behaviors. (**A**) The average frequency of each parental behavior for each group. (**B**) The average time spent engaged in each parental behavior for each group. (**C**) The average frequency of each non-parental behavior for each group. (**D**) The average time spent engaged in each non-parental behavior for each group. Whiskers indicate min. and max values. Solid lines indicate median values. N = 7–9 per group. * indicates *p* < 0.05 and ** indicates *p* < 0.01.

During the pup exposure test, both “Mother” and “Father” groups displayed similar levels of parental behaviors, including nest-building, carrying pups, licking/grooming pups, and huddling behavior. Sexually-naïve (virgin) males were divided into two groups—“Parental” or “Attacker”-based on their behavioral responses towards pups. The latency to attack for each “Attacker” male is included in Supplemental Table 1. Among the four types of parental behaviors collected, “Parental”, “Father”, and “Mother” groups were similar to each other, except that ”Parental” voles engaged in fewer instances (p = 0.006; Fig. 1A) and shorter durations (p = 0.027; Fig. 1B) of huddling behavior than “Mother” voles, but not “Father” voles. Although the “Parental”, “Father”, and “Mother” groups were generally comparable in the non-parental behaviors, particularly auto-grooming and locomotion, we found the “Parental” males rested more often (p = 0.009; Fig. 1C) and had a trend to spend more time sniffing (p = 0.054, ns) and resting (p = 0.051, ns) (Fig. 1D). The full details of the behavioral analyses are available in Supplemental Table 1.

### Transcriptome

**Figure 2:**
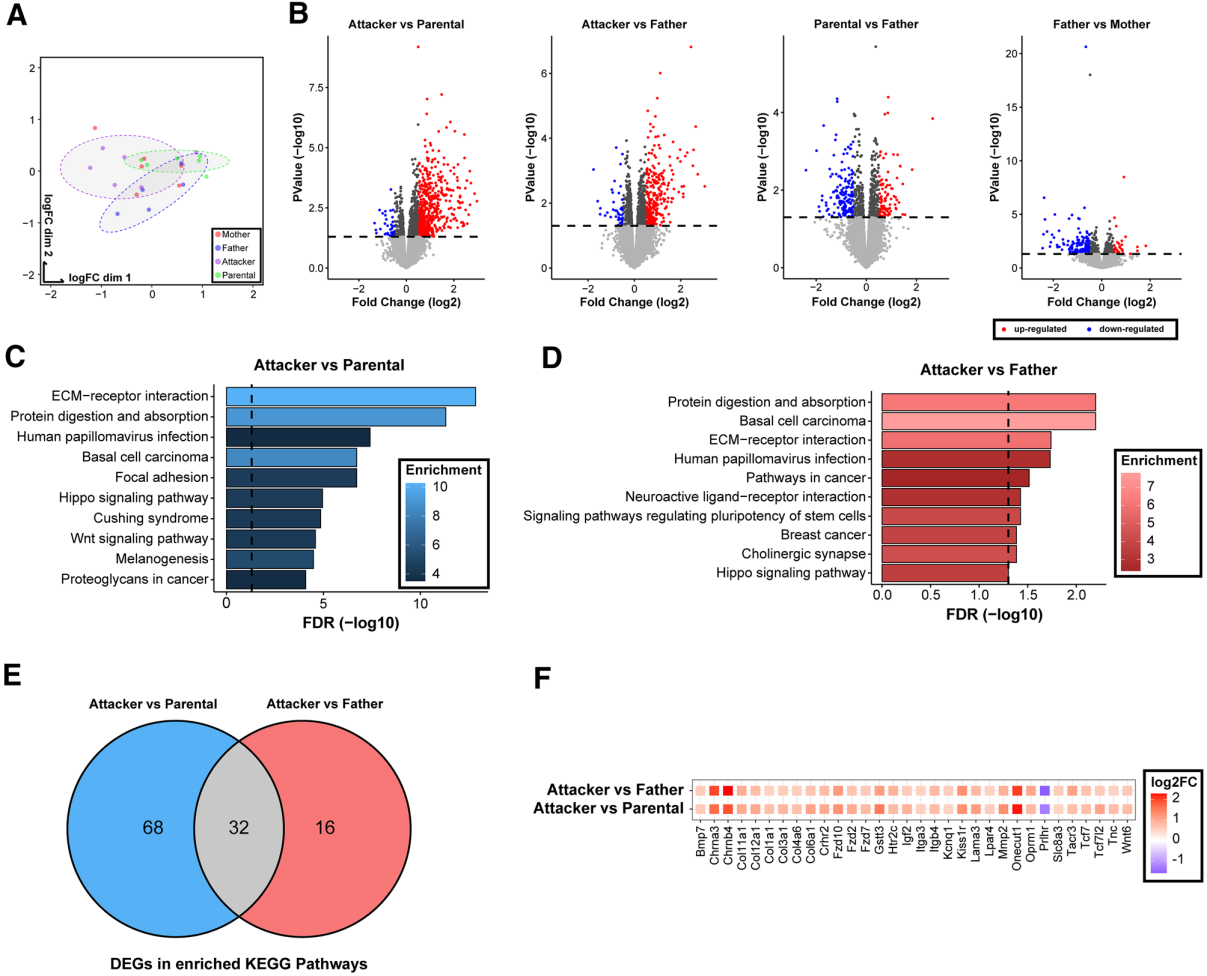
Hippocampal dentate gyrus transcriptome analysis. (**A**) Principal component analysis plot showing the variability of 23 RNA-seq libraries across all groups (6 Attacker, 6 Parental, 5 Father, 6 Mother). Points are color coded according to experimental group, and color-coded circles were drawn using the “stat_ellipse” function in ggplot2 to help represent each of the male groups. (**B**) Volcano plot visualizing differentially expressed genes, in log_2_ transformed Fold Change (log_2_FC) on the x-axis, with − log_10_ adjusted p values on the y-axis. Up-regulated and down-regulated genes are represented in red and blue, respectively, in the comparisons of “Attacker” vs “Parental”, “Attacker” vs “Father”, “Parental” vs “Father”, and “Father” vs “Mother”, as shown from the left to the right. The dashed line represents –log_10_(0.05). (**C**,**D**) Top ten significantly enriched KEGG pathways resulting from the “Attacker” vs “Paternal” comparison (**C**) and the “Attacker” vs “Father” comparison (**D**). The color gradient for each bar represents the enrichment ratio (observed/expected) with brighter color showing higher enrichment. The x-axis represents the − log_10_ transformed FDR value. (**E**) Venn diagram showing the number of differentially expressed genes in the enriched KEGG pathways for “Attacker” vs “Paternal” comparison (blue) and “Attacker” vs “Father” comparison (red), with the grey showing the union of the 32 overlapping genes between the two datasets. These overlapping genes are presented in a dotplot in (**F**) with color code assigned to represent the log_2_FC from the RNAseq comparison.

We found numerous DEGs in the four pair-wise comparisons of “Attacker” vs “Parental”, “Attacker” vs “Father”, “Parental” vs “Father”, and “Father” vs “Mother” (Fig. 2A,B, Table S2). In the PCA analysis (Fig. 2A), it appears that the “Mother” group mainly contributes to clouding of the representation, whereas each of the male experimental groups (“Father”, “Parental”, “Attacker”) appears to occupy their own graphic region with a little overlap. Though there are relatively fewer gene expression changes when comparing across pair-bonding experience or biological sex (N = 252 in “Parental” vs “Father”, N = 214 in “Father” vs “Mother”, Fig. 2B, Table S2), we found higher numbers of differentially expressed genes when “Attacker” group is compared to “Parental” or “Father” group (N = 553 and 352, respectively, Fig. 2B, Table S2), which indicates a more deviated DG transcriptome in “Attacker” virgin males, compared to the other groups of voles. Furthermore, both comparisons yielded more up-regulated genes (“Attacker” vs “Parental”: N = 518, “Attacker” vs “Father”: N = 305. Table S2) than down-regulated ones (“Attacker” vs “Parental”: N = 35, “Attacker” vs “Father”: N = 47; Table S2). We then investigated the over-represented KEGG pathways to obtain biological insights of the transcriptome change. We found the largest number of overrepresented pathways (N = 22) in the “Attacker” vs “Parental” comparison, and a moderate number of enriched pathways in the “Attacker” vs “Father” or “Parental” vs “Father” comparison (N = 10, or 4, respectively) (Table S3). In contrast, we did not identify over-represented pathways in the “Father” vs “Mother” comparison. Though generally more enriched in the “Attacker” vs “Parental” comparison, a number of these pathways are shared between the “Attacker” vs “Parental”, and “Parental” vs “Father” comparisons, such as “ECM-receptor interaction” and “protein digestion and absorption” (Fig. 2C,D, and Table S3). This suggests their expression changes may be implicated in parental behavioral variations in males. Furthermore, a set of genes that includes *Fzd*, *Tcf* and *Wnt* members were consistently detected in the enriched pathways specific to the “Attacker” vs “Parental” and “Attacker” vs “Father” comparisons, but not the other comparisons (Table S3). Though these genes are primarily Wnt signaling molecules, they may also be associated with other KEGG enriched pathways (e.g., Hippo signaling, cancer related pathways; Fig. 2C,D; Table S3). Furthermore, we found the overlapping pathways between the “Attacker” vs “Parental” and “Attacker” vs “Father” comparisons do not include the exact same DEGs. There are 32 overlapping DEGs shared within the overlapping KEGG pathways between the two analyses (Fig. 2E). Among them, many are selectively enriched in a few pathways that include Wnt signaling. For example, *Tcf7* and *Tcf7l2*, transcription factors modulating canonical Wnt signaling pathway output, are both differentially expressed within the “Attacker” vs “Parental” (*Tcf7l2*: log_2_FC = 1.06, p value = 6.81 × 10–5; *Tcf7*: log_2_FC = 0.77, p value = 0.0146, Fig. 2F, Table S2) and “Attacker” vs “Father” comparisons (*Tcf7l2*: log_2_FC = 0.635, p value = 0.0239; *Tcf7*: log_2_FC = 0.712, p value = 0.037, Fig. 2F, Table S2). We also found the main target for the canonical Wnt and BMP signaling *Lef1* is up-regulated in both comparisons (*Lef1*: log_2_FC = 0.563, p value = 0.011, “Attacker” vs “Parental”; *Lef1*: log_2_FC = 0.489, p value = 0.037, “Attacker” vs “Father”, Fig. 2F, Table S2). Together, these results point to the contrasting transcription signatures associated with the paternal behavioral dichotomy seen in virgin male voles and suggests the involvement of selective biological pathways. To validate our RNAseq findings, we performed qPCR on twelve of the genes chosen from the list in Fig. 2F, which include multiple Wnt signaling genes and several neural function related molecules (Supplemental Fig. 1). In general, majority of the genes tested had consistent changes in expression as was found in the RNAseq data. Among the twelve genes, ten (*Chrna3*, *Chrnb4*, *Col12a1*, *Fzd7*, *Fzd10*, *Igf2*, *Kissr1*, *Oprm1*, *Prlhr,* and *Tcf7l2*) were confirmed for their differential expression between “Attacker” vs “Parental”, eleven (*Chrna3, Chrnb4, Col12a1, Fzd7, Fzd10, Igf2, Kissr1, Oprm1, Prlhr, Tcf7l2, and Wnt6*) were confirmed for their transcription changes between “Attacker” vs “Father”. Among them, consistent with the RNAseq results, *Prlhr* was the only one down-regulated in both comparisons. In addition, all except *Tcf7l2,* were confirmed for no expression change between “Father” vs “Parental”.

**Figure 3:**
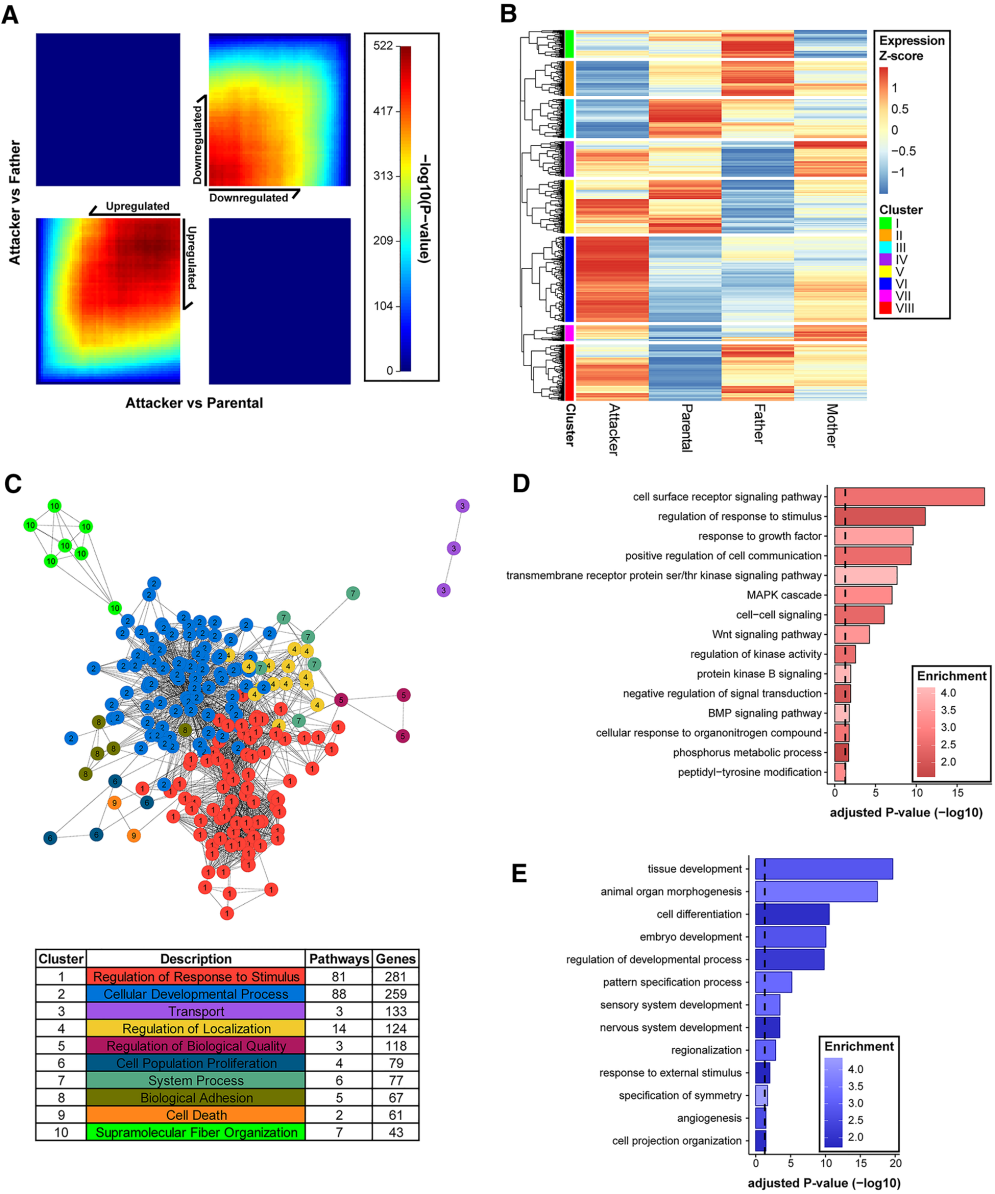
A more deviated transcriptome in Attacker virgin males. (**A**) RRHO analysis of all expressed genes in the “Attacker” vs “Parental” and “Attacker” vs “Father” comparisons, demonstrating either a discordant (top left and bottom right quadrants), or a concordant relationship (top right and bottom left quadrants). The overlap is made in a whole-transcriptome and threshold-free manner, where each pixel represents an overlap of the ranked lists. The color of each pixel represents the Benjamini–Yekutieli adjusted − log_10_(p value) of a hypergeometric test, with warmer colors reflecting more significance. (**B**) Clustered heatmap of all differential genes in each of the four comparisons. The analysis was done by the “pheatmap” software, with rows representing z-scores of normalized gene expression among the four groups in the analysis. The eight clusters (I–VIII) were generated using k-means clustering based on the normalized z-score of genes. (**C**) GOMCL cluster analysis of genes in heatmap cluster VI (**B**). Significantly over-represented biological process gene ontologies were represented in 10 clusters with reduced redundancy. The chart describes the ten simplified clustered pathways with the respective number of pathways included and the number of unique genes within each pathway to the right. Representative GO Biological Process Pathways of GOMCL cluster 1 and cluster 2 are displayed in (**D**,**E**), respectively. The over-represented pathways were modeled using the hypergeometric test with the whole annotated transcriptome as background. The color gradient of each bar represents the enrichment ratio (observed/expected), with brighter color showing higher enrichment. The x-axis represents − log_10_ transformed FDR values.

To further characterize the “Attacker” transcriptome, we performed RRHO^47,48^, an unfiltered transcriptome analysis, and found a vast degree of concordant signals between the “Attacker” vs “Parental” and “Attacker” vs “Father” transcriptome comparisons (total 3,723 genes up-regulated, 4,392 genes down-regulated, unfiltered analysis, Fig. 3A; Table S4). However, virtually no discordant signal was detected in the same analysis (only 1 gene that is down in “Attacker” vs “Parental” comparison is up in “Attacker” vs “Father” comparison; and no overlap between up-regulated genes in “Attacker” vs “Parental” comparison and down-regulated genes in “Attacker” vs “Father” comparison; Fig. 3A; Table S4). This further supports the notion of a profoundly contrasting transcriptome in “Attacker” virgin males when compared to “Parental” and “Father” groups, which shared broad similarities.

To obtain a genomic scale overview of transcriptome changes across all groups, we carried out a nuanced approach by constructing a “Differential Expression Clustering” heatmap analysis that includes all DEGs from any of the four comparisons (i.e., “Attacker” vs “Paternal”, “Attacker” vs “Father”, “Paternal” vs “Father”, “Father” vs “Mother”; Table S2) through plotting their inter-group gene expression z-scores (Table S5). Among the eight clusters (I–VIII) classified through k-means clustering, cluster VI has the highest number of DEGs. They were up-regulated in “Attacker”, which is more discordant from the other three groups that were generally down-regulated (Fig. 3B). As cluster VI genes were over-represented in numerous biological process pathways (N = 240 over-represented pathways, Table S6), we further performed a “Gene Ontology Clustering” analysis to construct a clustered similarity network with the majority of these over-represented pathways included (N = 213) to facilitate our interpretation (Fig. 3C, Table S7). Of the ten clusters (named in Arabic numbers 1 to 10) we derived, clusters 1 and 2 contained the largest set of biological process pathways and the highest number of genes (Cluster 1 = 81 pathways, 281 genes; Cluster 2 = 88 pathways, 259 genes, Fig. 3C, Table S7). Together, clusters 1 and 2 account for close to 80% of biological pathways and about 43% of DEGs in differential expression cluster VI. Within cluster 1 (Fig. 3D, Table S7) that is represented by the parent GO term “regulation of response to stimulus”, we found an enrichment of several signaling pathways, with some also recognized within the aforementioned KEGG pathway analysis (Fig. 2), such as Wnt signaling (Enrichment = 3.42, adjusted p value = 0.00128, Fig. 3D and Table S7). For cluster 2 analysis that is represented by the parent GO term “cellular developmental process”, a number of development pathways are enriched, such as nervous system development (Enrichment = 1.851, adjusted p value = 0.000349, Fig. 3E, Table S7), both positive and negative regulation of cell-differentiation (positive regulation: Enrichment = 2.259, adjusted p value = 0.0123; negative regulation: Enrichment = 2.85, adjusted p value = 4.55 × 10^–5^, Table S7). This may reflect DG’s role in adult neurogenesis^62^ that contributes to the paternal behavioral dichotomy.

Beyond cluster VI, in the “Differential Expression Clustering” heatmap (Fig. 3B), clusters II and III also displayed an opposing transcription pattern in “Attacker” compared to the other three groups, with genes in “Attacker” group being down-regulated. From them, numerous gene ontology terms were over-represented, which included neurogenesis (Enrichment = 2.67, adjusted p value = 9.02 × 10^–5^), and synaptic signaling (Enrichment = 5.65, adjusted p value = 7.48 × 10^–10^, Table S6), two of the top enriched ontology terms.

Looking at these gene expression clusters showing up- and down-regulation of neurogenesis related GO terms, this indicates a dysregulation of hippocampal neurogenesis instead a sole up-regulation or down-regulation itself. Furthermore, basal Wnt signaling in the adult brain is necessary to maintain synaptic connectivity, where constitutive release of Wnt ligands contributes to neuronal network maintenance by affecting synaptic mechanisms. Outside of basal Wnt signaling, there appears to be activity-dependent mechanisms to refine, by either strengthening or weakening, synaptic connections^63^, which is consistent with our findings in both directions of synaptic signaling related changes. Together, our results demonstrate a unique biological signaling signature of the DG transcriptome associated with the paternal behavioral dichotomy.

### DNA methylome

**Figure 4:**
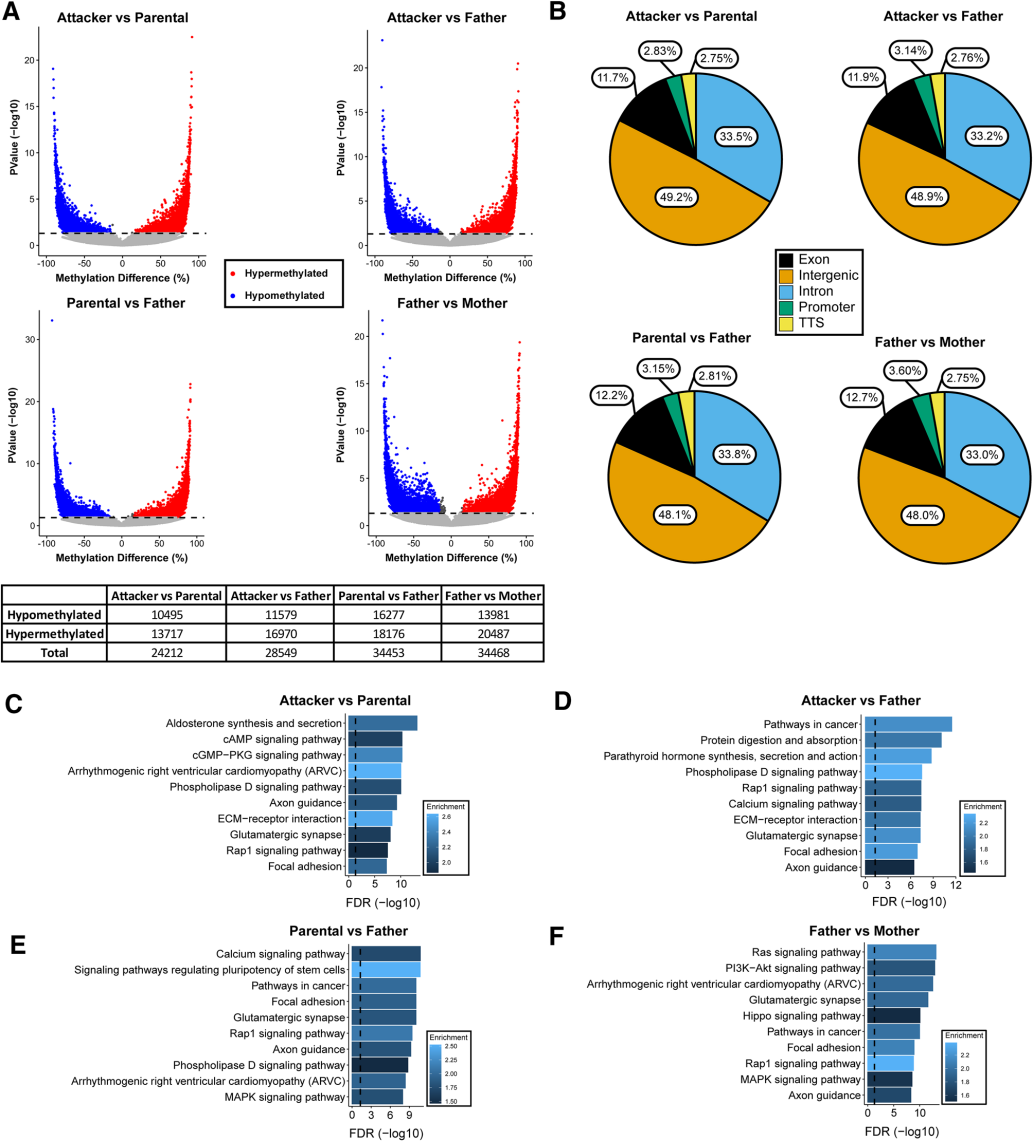
Differential DNA methylation analysis. (**A**) Volcano plot visualizing DMCs. Percentage of methylation difference is represented on the x-axis, while the − log_10_(p value) is represented on the y-axis. Hypermethylated CpGs and hypomethylated CpGs are represented in red and blue, respectively. The horizontal dashed line corresponds to − log_10_(0.05) to represent a threshold for significance. The chart on the bottom demonstrates the numbers of differentially methylated sites in each comparison. (**B**) Pie charts represent the genomic feature distribution of the DMCs from each comparison. Labels within each section represent the % of DMCs that fall within these features. TTS stands for transcription termination site. (**C**,**D**,**E**,**F**) The top 10 ranked over-represented KEGG pathways of genes that contain DMCs within their boundary (from 2000 bp upstream of the transcription start site to 1000 bp downstream of the transcription termination site) for the comparisons of “Attacker” vs “Parental” (**C**), “Attacker” vs “Father” (**D**), “Parental” vs “Father” (**E**), and “Father” vs “Mother” (**F**). In each of the panels, the vertical dashed line represents an FDR cutoff of 0.05, and the color represents the enrichment ratio of the pathways.

To obtain a molecular insight of parental behavior beyond the transcriptome, we examined DNA methylation in the DG, which may mediate gene expression. By using RRBS methylome profiling^34^, we found numerous DMCs in each of the four pair-wise comparisons with consistently more hypermethylation sites than hypomethylation sites (Fig. 4A, Table S8). Unlike what we found in the transcriptome analysis that “Attacker” vs “Parental” and “Attacker” vs “Father” comparisons had the most transcription changes than the other comparisons, the highest number of DMCs was found in “Parental” vs “Father” and “Father” vs “Mother” comparisons (total number in 34,453, and 34,468, respectively). In contrast, “Attacker” vs “Parental” and “Attacker” vs “Father” comparisons have substantially lower number of methylation changes (24,212 and 28,549 DMCs, respectively; Fig. 4A, Table S8). This may be explained by the plausible involvement of DNA methylation in sex difference or sexual experience. In addition, we calculated DMRs from the DMCs for each comparison. In line with our DMCs analysis, we found that the “Parental” vs “Father” and “Father” vs “Mother” comparisons had more DMRs (with 322 and 340 DMRs, respectively), than “Attacker” vs “Parental” and “Attacker” vs “Father” comparisons (with 161 and 224 DMRs, respectively, Table S8). The small number of DMRs could be explained by the enrichment methods from the RRBS library preparation and influenced by the cell type heterogeneity in the DG, therefore we opted to focus primarily on DMCs for further analyses.

We found that most of the DMCs are located in intergenic and intronic genomic regions (around 50% and 30%, respectively, in each comparison; Fig. 4B, Table S8), and around 3% of DMCs reside are at promoters (Fig. 4B). In order to determine whether the possible captured CpG sites in the reference genome were influencing the genomic feature distribution, we calculated the proportion of CpG sites within 100 bp of all MspI sites in the reference genome. After performing Hypergeometric tests, we found that there was an over-representation of DMCs in exon (log_2_ Odds Ratio (log_2_OR) = 0.297, FDR = 2.17 × 10^–215^, “Attacker” vs “Parental”; log_2_OR = 0.308, FDR = 2.84 × 10^–277^, “Attacker” vs “Father”; log_2_OR = 0.315, FDR = 1.21 × 10^–244^, “Parental” vs “Father”; log_2_OR = 0.337, FDR = 4.18 × 10^–287^, “Father” vs “Mother”; Table S9) and TTS regions (log_2_OR = 0.148, FDR = 1.17 × 10^–16^, “Attacker” vs “Parental”; log_2_OR = 0.149, FDR = 9.94 × 10^–20^, “Attacker” vs “Father”; log_2_OR = 0.157, FDR = 1.21 × 10^–26^, “Parental” vs “Father”; log_2_OR = 0.149, FDR = 3.35 × 10^–23^, “Father” vs “Mother”; Table S9). Furthermore, we found that there was a significant under-representation of promoter DMCs (log_2_OR = − 0.535, FDR = 1.39 × 10^–230^, “Attacker” vs “Parental”; log_2_OR = − 0.489, FDR = 5.00 × 10^–241^, “Attacker” vs “Father”; log_2_OR = − 0.487, FDR = 3.26 × 10^–289^, “Parental” vs “Father”; log_2_OR = − 0.429, FDR = 9.63 × 10^–242^, “Father” vs “Mother”; Table S9) with respect to the reference distribution. Because we have defined promoter from − 2000 bp to the start of TSS, instead of into the exon 1 as some others have done, this may be contributing to decreased promoter representation and increased exon representation.

To explore the functional implication of this vast array of DNA methylation changes, we examined the pathways over-represented by genes containing DMCs in promoter or gene body regions (Table S10). We found that over 100 KEGG pathway terms are enriched in each of the four comparisons and many of them are overlapping. By examining the top 10 enriched pathways (Fig. 4C–F), none appears to be unique to each comparison. Instead, over 80 pathways are over-represented in all four comparisons (Fig. S2A, Table S10). These include “ECM-receptor interaction” and “protein digestion and absorption”, the top two common pathways identified in the aforementioned transcriptome comparisons (Fig. 2C,D; Table S3), which suggests a canonical transcription regulatory role of DNA methylation in paternal behavior. “Oxytocin signaling”, a key pair-bonding pathway that is known to subject to DNA methylation modulations^23^, is also enriched in all comparisons (Fig. S2A). Additionally, “Rap1 signaling”, “glutamatergic synapse”, “axon guidance”, “PI3K-Akt signaling”, “calcium signaling”, “cAMP signaling”, “cholinergic synapse”, “Ras signaling”, “GnRH signaling”, “thyroid hormone synthesis” are all notable overlapping pathways, to name a few (Fig. S2A). Furthermore, we found 60 pathways over-represented in one, two, or three, but not four, comparisons (Fig. S2B, Table S10), with over half of them not enriched in the “Attacker” vs “Parental” comparison, such as “neurotrophin signaling pathway” and “VEGF signaling pathway”. Among them, a dozen pathways are only enriched in one comparison (e.g., P53 signaling in “Attacker” vs “Father”, vasopressin in “Father” vs “Mother”), but their enrichments are not the highest based on FDR values (Fig. S2B). In the meantime, we found some immune related pathways appearing to be enriched in “Father” related comparisons, which include “NF-kappa B signaling”, “T cell receptor signaling”, “B cell receptor signaling”, “Fc signaling”, to name a few (Fig. S2B).

### Transcriptome–methylome correlation

The numerous differential methylation pathways that are mostly overlapping across comparisons may suggest DNA methylation’s canonical roles widely associated with parental behaviors. Therefore, the DNA methylation changes may represent a spectrum of molecular signatures of the parental behavior variations as indicated by the inter-group behavioral examinations (Fig. 1). Given the regulatory role of DNA methylation in gene transcription, we then moved forward to explore any association between DNA methylome and transcriptome alterations.

We began our study on the interplay between the transcriptome and DNA methylome in the prairie vole DG by examining the changes in a gene's expression and the differential methylation of cytosines within the same gene locus. Our preliminary analysis indicated no direct correlation between these two data sets. Consequently, we chose to divide our dataset into quantiles based on gene expression and analyze the differential methylation of cytosines corresponding to various degrees of gene expression in either gene-body regions or promoter regions separately. We initially noticed several intriguing qualitative observations (Fig. S3A–H). First, we noticed that for quantiles related to the most decreased gene expression, the average differential methylation change for cytosines associated with gene promoters was positive (Fig. S3B,D,F,H). This observation seemed to align with the traditional gene silencing role of DNA methylation. Secondly, we observed variability in corresponding DNA methylation changes for DMCs located both in the gene-body and the promoter regions for genes with medium-level expression quantiles (Fig. S3A–H). This observation could potentially represent a mix of dynamic gene regulatory mechanisms influencing variable gene expression. Lastly, we observed that in the highest gene expression quantiles, the associated changes in DNA methylation for DMCs within these genes also tended to be positive (Fig. S3A–H). However, when we correlated the gene expression quantiles with their associated DNA methylation levels using Spearman correlations, no significance was detected. Only two quantiles showed a trend towards significant relationship between methylation and expression changes after adjusting for multiple comparisons using a Bonferroni-corrected α value. For example, in the gene-body subset of the “Parental” vs “Father” comparison, there was a trending positive correlation between gene expression and DNA methylation for quantile 8 (rho = 0.253, p value = 0.011, Bonferroni-α = 0.0064, Fig. S3I, Table S11), and in the promoter-focused “Attacker” vs “Father” comparison, there also was a trending positive correlation between gene expression and DNA methylation in quantile 3 (rho = 0.683, p value = 0.014, Bonferroni-α = 0.0127; Fig. S3J, Table S11).

While these quantiles were not significantly correlated with DNA methylation, we recognized a number of functional meaningful genes, which included those belonging to the Wnt signaling pathway. For example, *Dkk3*, a secreted inhibitor of Wnt signaling, was slightly under-expressed while having a hypermethylated CpG in the promoter region of “Attacker” (log_2_FC = − 0.264, differential methylation = 0.743, “Attacker” vs “Parental”, Table S11), which is consistent with the repressive role of DNA methylation on gene transcription at gene promoters. Furthermore, *Fzd10*, a membrane bound Wnt receptor, was up-regulated with a hypomethylated CpG within intron 1 (log_2_FC = 1.280, differential methylation = − 0.613, “Attacker” vs “Parental”, Table S11). In addition, there were two gene body hypermethylated sites in the upregulated gene *Enpp2* (log_2_FC = 1.862, “Attacker” vs “Parental”, Table S11), whose protein product can function as a phosphodiesterase to catalyze the production of lysophosphatidic acid (LPA), which is known to activate the β-catenin pathway^64,65^. Together these results implicate that DNA methylation selectively impacts certain regulatory systems to affect paternal behaviors.

**Figure 5:**
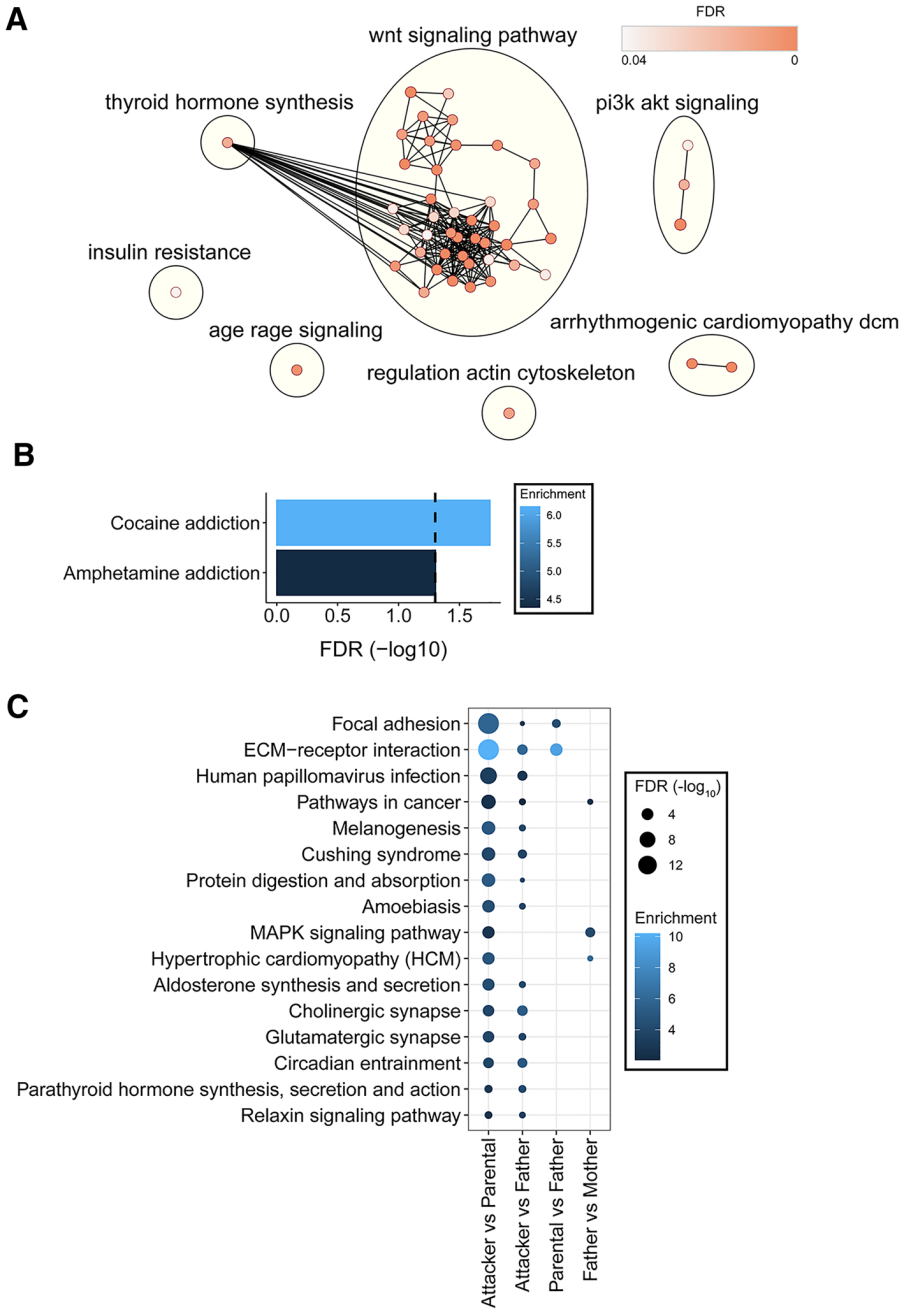
Pathway analysis of genes with both transcription and methylation changes. (**A**) The unique over-represented KEGG pathways in the “Attacker” vs “Parental” comparison. KEGG Pathway analysis was done after clustering using an affinity propagation algorithm implemented in the Cytoscape tool. Seven main functional clusters (e.g., Wnt signaling pathway) were identified (yellow circles), each containing one or more connected KEGG terms (nodes). The color of each node represents the FDR value from the over-representation analysis, while the edges of the network represent similarity between the clustered KEGG pathways. (**B**) The unique over-represented KEGG pathways in the “Attacker” vs “Father” comparison. The vertical dashed line represents FDR of 0.05, while the color represents the enrichment ratio of the over-representation test. (**C**) The shared over-represented KEGG pathways from the comparisons listed on the x-axis. Size of each dot represents the − log_10_ transformed FDR values, while the color represents the enrichment ratio from the over-representation test. The missing datapoints show that the respective pathway was not found to be over-represented in the comparison listed.

To obtain a systemic biological interpretation of the genes that have both transcription and DNA methylation changes, we analyzed the KEGG pathways over-represented by these genes (Fig. 5, Table S12). We found the highest number of enrichment terms in the “Attacker” vs “Parental” comparison (N = 65), followed by the “Attacker” vs “Father” comparison (N = 16, Fig. 5A,B, Table S12). In contrast, in “Parental” vs “Father” and “Father” vs “Mother” comparisons, only two and three pathways were enriched, respectively, and none of them was unique to any comparison (Fig. 5C, Table S12). These suggest that DNA methylation may play a main regulatory role on gene expression associated with the aggressive paternal behavior. Moreover, among the 65 enrichment terms in the “Attacker” vs “Parental” comparison, 49 pathways were not over-represented in other comparisons (Fig. 5A, Table S12), which may indicate their unique contributions to paternal behavioral dichotomy in virgin male voles. Using affinity propagation clustering, implemented in the Cytoscape EnrichmentMap plugin^58–60^ further illustrates that the majority of these pathways (N = 40) are highly connected under one single main functional cluster “Wnt signaling pathway”, which also has a substantial overlap with “Thyroid hormone synthesis” (Fig. 5A, Table S12). Although the “Father” group voles have a more similar parental behavioral phenotype to the “Parental” group (Fig. 1), who also share an overall more similar transcriptome when compared to the “Attacker” virgin voles (Figs. 2, 3), the KEGG pathway analysis on genes with both transcription and DNA methylation changes in the “Attacker” vs “Father” comparison did not lead to any many unique pathways as in the “Attacker” vs “Parental” comparison. Instead, only two pathways were uniquely enriched in the “Attacker” vs “Father” comparison, with both of them addiction related (“cocaine addiction” and “amphetamine addiction”, Fig. 5B, Table S12). In the meantime, we found a majority of the pathways shared in two or more comparisons (11 out of 16, Fig. 5C) were only over-represented in the “Attacker” vs “Parental” and “Attacker” vs “Father” comparisons.

## Discussion

In this study, we examined the dentate gyrus (DG) transcriptome and methylome to obtain molecular insights of parental behaviors of prairie voles following a pup-exposure paradigm. In this behavioral model, both father and mother voles display biparental care, whereas virgin male voles demonstrate parental behavioral dichotomy with some behave parentally and the rest show aggression. Though the prairie vole genome is still under construction with large portions of chromosomes isolated on independent genome scaffolds which poses a challenge for genomic research, our exploratory study has led to some interesting findings that support a DNA epigenetics based molecular underpinning of paternal behavioral dichotomy.

We found that spontaneously aggressive virgin male prairie voles (“Attacker”) exhibited unique molecular signatures (both transcriptome and methylome changes) compared to spontaneously parental virgin male voles (“Paternal”). Though variable gene transcription patterns have been reported in other brain regions^66^, we found profound genome-wide gene expression changes in the DG of “Attacker” virgin males, when compared to either “Parental” or “Father” voles, which were more comparable. The differential genes from these analyses are selectively enriched in a number of biological pathways that suggest their functional implications in parental behavior. Notably, we found expression changes of several Wnt signaling molecules that are associated with the parental behavior variance. Wnt signaling is a highly conserved pathway that plays fundamental roles in development and homeostasis^67–70^. Accumulating evidence supports its major roles in neural development, synaptic plasticity, as well as brain diseases^71^. Wnt signaling generally includes the canonical and noncanonical pathways. The canonical pathway is usually referred as the “Wnt/β-catenin pathway” due to its dependence on the stabilization of β-catenin. The binding of Wnt ligands to the cell surface Frizzled (Fzd) receptors and LRP5/6 co-receptors will endocytose this complex and inhibits the β-catenin destruction complex that consists of GSK-3β. This leads to increased levels of β-catenin and its translocation to the nucleus to mediate gene expression through the interaction with TCF/LEF transcription factors. In addition, the silencing of the canonical pathway may be carried out through the activation of the β-catenin destruction complex by Wnt/β-catenin inhibitor DKK. In contrast, the non-canonical Wnt signaling pathway functions without β-catenin or GSK-3β as an intermediate molecule. It is triggered by Wnt ligand binding to the Fzd receptor and coreceptors, which then activates either the Wnt/PCP (planar cell polarity) (also named Wnt/JNK) pathway or the Wnt/calcium pathway, two main downstream branches of the non-canonical Wnt signaling. The non-canonical pathways play significant roles in cytoskeleton remodeling, synaptic plasticity, and axon guidance^71^. The identification of various Wnt signaling molecules (e.g., *Wnt*s, *Fzd*s, *Dkk*, *Tcf/Lef*s), and the enrichments of related pathways (e.g., axon guidance, calcium signaling, synaptic signaling, etc.) in our differential transcriptome analyses suggest a broad implication of Wnt signaling in parental behavior modulation that likely includes both canonical and non-canonical pathways. Given the dentate gyrus is a major adult neurogenesis site and Wnt signaling’s involvement in both neurogenesis^72,73^ and mature brain synaptic plasticity, it remains to be addressed at what stage Wnt signaling is involved in parental behavior.

As social encounters can induce stress responses which, in turn, escalate aggression including infanticidal behaviors^74,75^ and the Wnt signaling pathway has been implicated in social behaviors^76,77^, we cannot exclude the possibility that the difference in Wnt signaling between the pup attackers and non-attackers may be related to their differences in stress responses and the subsequent pup-directed behaviors. However, studies from the monogamous rodent species, such as prairie voles and California mice, have indicated that acute stressful experience enhanced paternal behaviors and decreased pup attacks in males^78,79^. Therefore, the molecular differences we detected in association with paternal behaviors may be better explained by their responses to pup associated stimuli, rather than general stress reactions. In addition, we have primarily focused on the paternal behavior related analyses, which appear to be more significant. However, we think the “Mother” group is helpful in the behavioral analysis because voles are naturally biparental, having a comparison for father pup-care appeared necessary. In the meantime, we also found it is difficult to compare the “Mother” group with the two virgin male vole groups as there are many variables between them. One thing that would help in the future is to include a fifth group of virgin females.

In order to further evaluate the degree of gene expression changes in the whole transcriptome, we utilized a modification of a hypergeometric overlaps test RRHO. We found a great deal of similarities between the unfiltered transcriptome comparisons of “Attacker” vs “Parental” and “Attacker” vs “Father”, which provides a novel molecular basis of the paternal behavioral dichotomy. Notably, in this RRHO analysis, which was performed in a whole-transcriptome threshold-free manner, we found DNA epigenetic modification enzymes, *Dnmt3a* and *Tet1,* have concomitant transcription trends between “Attacker” vs “Parental” and “Attacker” vs “Father” comparisons (Table S4). DNA methyltransferases (DNMTs) catalyze DNA methylation through the covalent addition of a methyl group to cytosine nucleotide, which can be altered through a series of oxidation reactions mediated by Ten-eleven translocation (TET) methylcytosine dioxygenases, that may ultimately lead to unmethylated cytosines^80–82^. In the future, it will be interesting to identify if any DNA methylation enzyme has expression changes in specified cell types.

Though the studies of DNA methylation in prairie voles remain few in number, nearly all of them focused on individual genes, particularly nonapeptides (e.g., vasopressin and oxytocin)^20,83,84^. In our genome-wide DNA methylation profiling, we found the differential methylation loci associated genes are enriched in a broader range of KEGG pathways with many not represented in the differential transcriptome analysis. Compared to the transcriptome examination, epigenome profiling may demonstrate molecular changes in a multi-dimensional manner to reflect not only past experience, but also future inducibility. It may also capture alterations in subpopulations of cells that are below the detection threshold of a bulk tissue RNAseq analysis. Likely, some of the DNA methylation changes impact transcriptome outputs in a defined cell population that were unable to be identified in our whole DG tissue RNAseq examination. In addition, the RRBS sequencing we applied in this study only surveys a portion of the DNA methylome^85^. If the RRBS results reflect a representative DNA methylome overview, more widely distributed methylation changes are expected to occur across the whole genome. This will be interesting to explore in the future, which demands significant financial and bioinformatic endeavors. When we performed the methylome pathway analyses, we limited them to differentially methylated sites at gene promoters and gene bodies. The reason to exclude DNA methylation changes at distal intergenic regions, which account for a major portion of differentially methylated sites, is because it remains challenging to identify their target genes. Often times, the intergenic regulatory DNA elements bypass their nearby genes to modulate transcription of genes located a long linear distance away through three-dimensional looping^86^. How the higher order genome is organized in prairie vole DG is unknown.

While all above factors may explain the variable findings between our transcriptome and DNA methylome analyses, it is intriguing to see several overlapping pathways that are enriched with both DNA methylation and transcription changes, such as “ECM–receptor interaction” and “protein digestion and absorption”. This suggests DNA methylation may modulate parental behavior associated gene transcription changes in selective pathways. From this integrated analysis, we have noticed several interesting candidate pathways, such as cytoskeleton, cholinergic signaling, immune signaling, that deserve further investigation. Particularly, we have found that genes with both methylation and transcription changes in the “Attacker” vs “Parental” comparison are profoundly enriched in the “Wnt signaling” cluster (Fig. 5A). Though growing evidence has indicated Wnt signaling’s role in neural development and function in recent years, our integrated transcriptome and DNA methylome examinations suggest a novel function of Wnt signaling in parental behavior, particularly paternal behavioral dichotomy. Though still few in number, accumulated evidence in recent years reported Wnt signaling in the brain may be under epigenetic regulations, such as histone modifications and microRNA^77,87,88^. In a genome-wide DNA methylome profiling of PTSD patient blood, Wnt was among the top enriched signaling pathways with DNA methylation changes^89^. Notably, in the only study that has investigated DNA methylation in prairie vole brains at a genomic scale, epigenetic age was assessed through a custom array^90^. It was found that pair bonded voles have a younger brain. Interestingly, among the four genes that showed most age-related DNA methylation changes is *Fzd1*, a Wnt signaling gene.

In summary, our study has provided an unprecedented integrated view of dentate gyrus transcriptome and DNA methylome in prairie voles, which are associated with the parental behavior differences. The significant correlation between DNA methylation and gene expression in selective biological pathways illustrates a novel role of DNA methylation in parental behavior. By the high-throughput nature of these genomic approaches, our datasets provide a valuable reference that awaits further biological validations.

## Supplementary Information

Supplementary Information 1.

Supplementary Information 2.

Supplementary Information 3.

Supplementary Information 4.

Supplementary Information 5.

Supplementary Information 6.

Supplementary Information 7.

Supplementary Information 8.

Supplementary Information 9.

Supplementary Information 10.

Supplementary Information 11.

Supplementary Information 12.

Supplementary Information 13.

Supplementary Information list.

## Data Availability

Next generation sequencing files and processed data for both RNAseq and RRBS datasets is available at the NCBI Geo under the GSE214799 superseries.
