# Supplementary material for: Transcription and DNA methylation signatures of paternal behavior in hippocampal dentate gyrus of prairie voles
Source: Sci Rep. 2023 Jul 7;13:11020. doi: 10.1038/s41598-023-37521-2 (PMC10328943; doi:10.1038/s41598-023-37521-2)
Supplement: Supplementary file 1 — Supplementary Information 1. [file 41598_2023_37521_MOESM1_ESM.docx]

**Supplemental Figures**


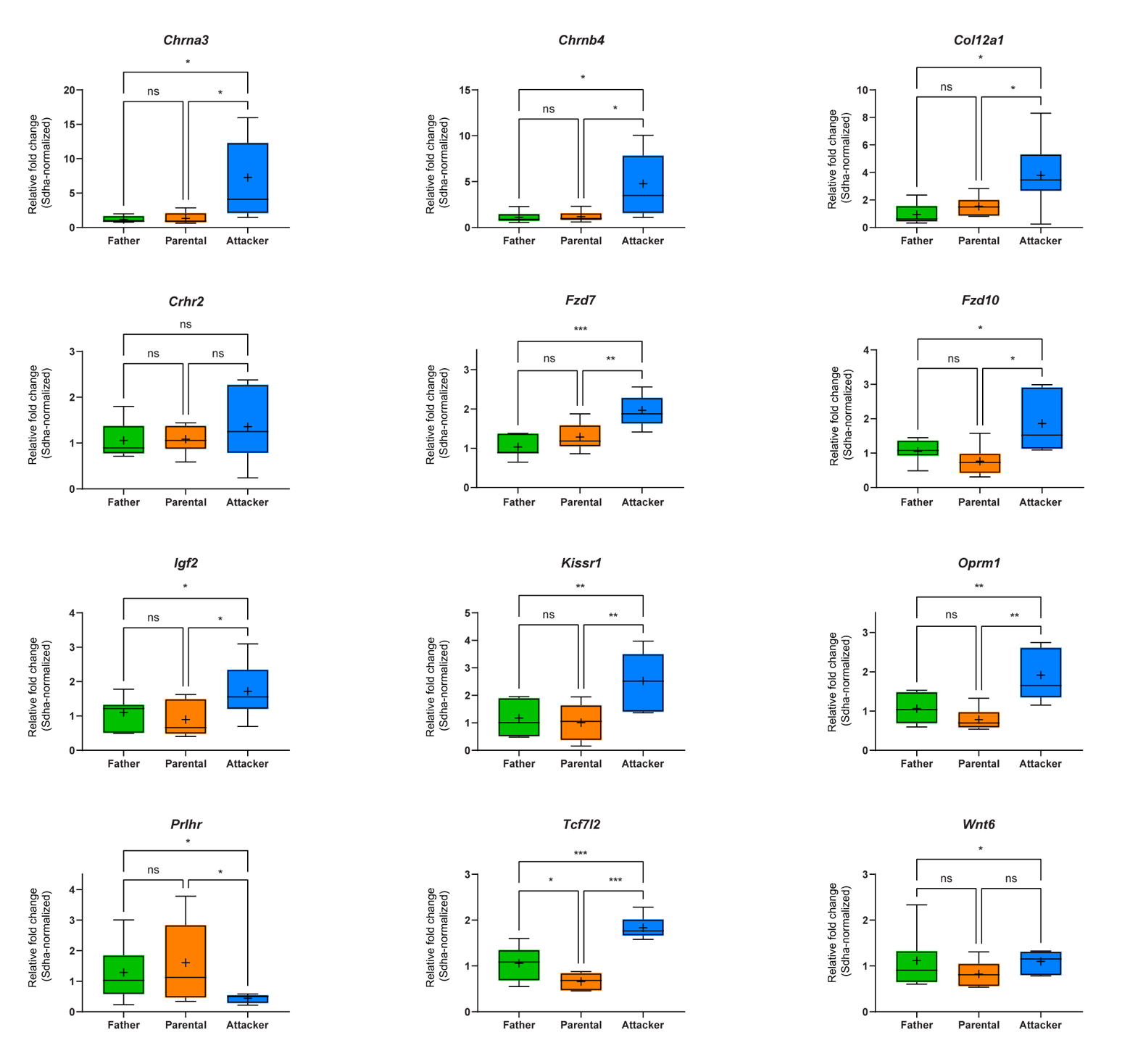


**Supplemental Figure 1: qPCR validation of RNAseq differentially expressed genes.** Box plots represent the fold change in gene expression for 12 candidate genes (*Chrna3*, *Chrnb4*, *Col12a1*, *Crhr2*, *Fzd7*, *Fzd10*, *Igf2*, *Kissr1*, *Oprm1*, *Prlhr*, *Tcf7l2*, *Wnt6*) in two distinct groups of sexually naïve males, “Parental” (orange) and “Attacker” (blue), compared to the “Father” group (green). Each box plot displays the median (indicated by the horizontal line within the box) and mean (marked by the '+' symbol) gene expression for each group. Gene expression values were normalized to *Sdha*. Statistical significance between groups was determined using one-tail Student's t-tests after variance assumption detected using F-tests, with the following p-value designations: *, p < 0.05; **, p < 0.01; ***, p < 0.001. “ns” denotes no statistical significance. N = 5-8/replicates per group.


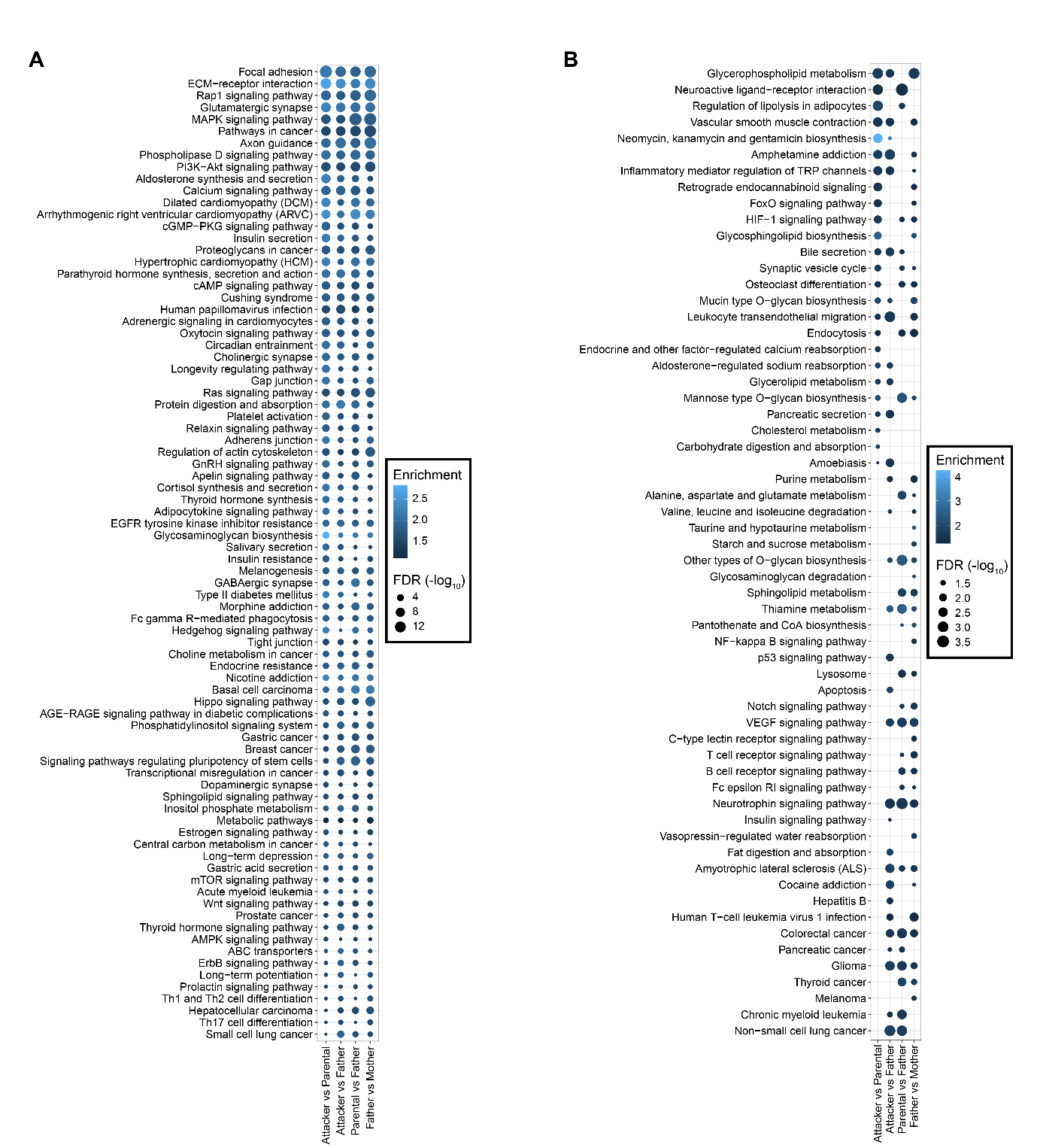


**Supplemental Figure 2:** **KEGG pathways of differentially methylated CpGs**. **A & B.** These dot plots show over-represented KEGG pathways from genes that contained a differentially methylated CpG site within their genic regions from each of the comparisons. This includes (-2000 bp upstream of the TSS to 1000 bp downstream of the TTS). The dot size represents the FDR from the over-representation test, while the color corresponds to the enrichment, or observed / expected values from the test. In **A**, pathways that were shared among all four comparisons are listed, while in **B**, pathways that were enriched in one to three comparisons.


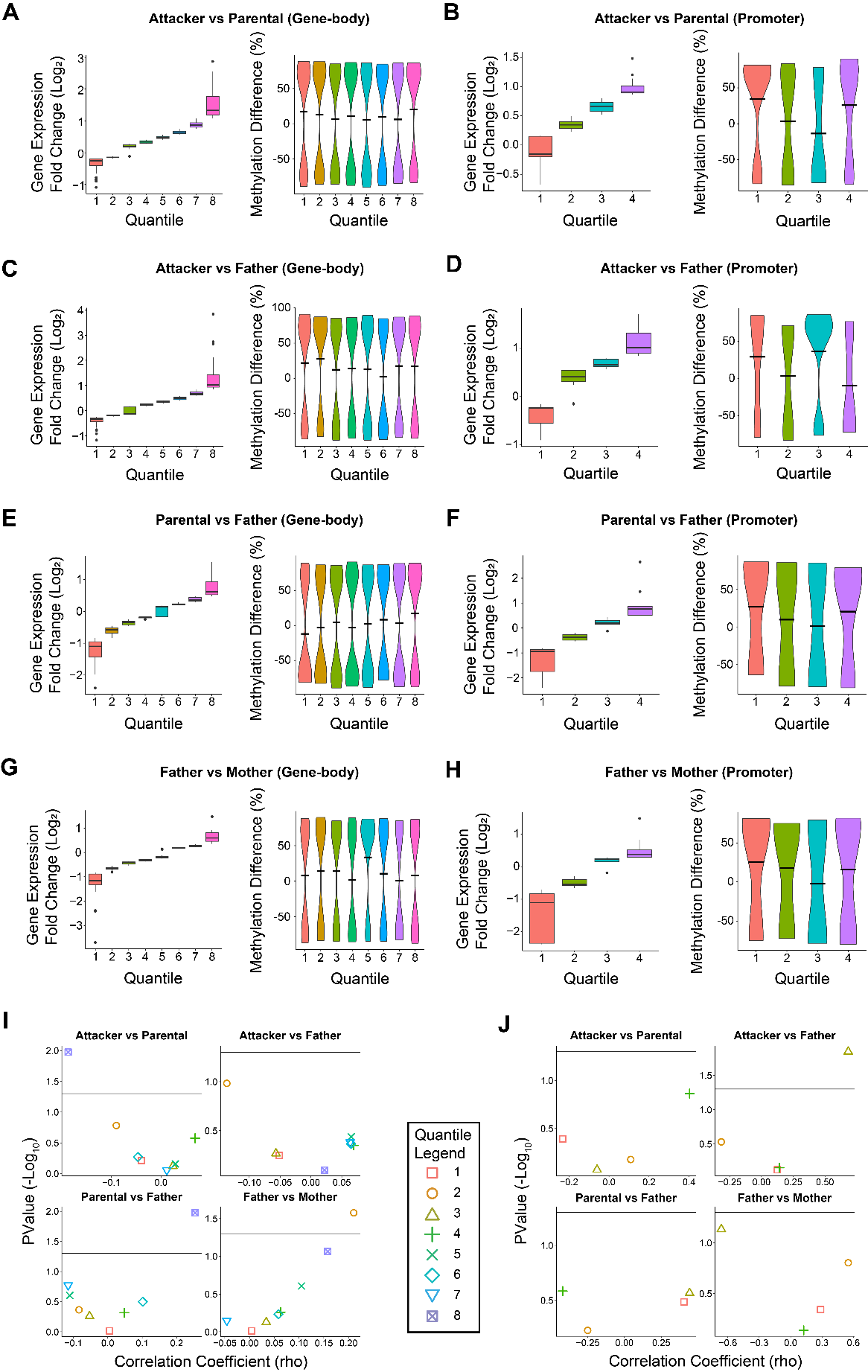


**Supplemental Figure 3: Correlations between gene expression and DNA methylation changes. A.** In “Attacker” vs “Parental” comparison, genes that have both differential expression and differentially methylated CpG sites within their gene boundary were divided into eight quantiles based upon the value of log_2_ fold gene expression change. The left plot represents the log_2_ fold change of the differentially expressed genes, with the horizontal line represents the median of each group. The right plot indicates DNA methylation differences of each of the eight quantiles. The width of the violin plot indicates the proportion of datapoints at y-axis values. The black line represents the mean value of the methylation differences. **B.** In “Attacker” vs “Parental” comparison, the analysis is done in the same manner as in **A** for differentially expressed genes that have DNA methylation changes in their promoters, except all genes were separated into 4 quartiles. For pairs **C** & **D, E** & **F**, and **G** & **H**: they represent the same type of analysis as in **A** & **B**, but for “Attacker vs Father”, “Parental vs Father”, and “Father vs Mother” comparisons respectively. **I** & **J.** A scatter plot of spearman correlation coefficients and the associated p-values from the correlation analysis of quantiles in the four comparisons of the analysis with their labels listed above each portion of the chart. The horizontal line represents -log_10_(0.05) as an uncorrected α-value. Panels in **I** correspond to the eight quantiles in **A**,**C**,**E**, and **G,** while panels in **J** correspond to the four quartiles in **B**,**D**,**F**,**H**. The horizonal lines in figures **I** & **J** represent an α-value of 0.05, however corresponding data in table S11 have Bonferroni corrected significance thresholds.
