# Supplementary material for: Transcription and DNA methylation signatures of paternal behavior in hippocampal dentate gyrus of prairie voles
Source: Sci Rep. 2023 Jul 7;13:11020. doi: 10.1038/s41598-023-37521-2 (PMC10328943; doi:10.1038/s41598-023-37521-2)
Supplement: Supplementary file 14 — Supplementary Information list. [file 41598_2023_37521_MOESM14_ESM.docx]

**List of Supplementary Materials**

Supplemental Table 1: Behavioral analysis

Supplemental Table 2: Differentially expressed genes

Supplemental Table 3: Over-represented KEGG pathways from DEGs

Supplemental Table 4: RRHO gene list

Supplemental Table 5: Normalized gene counts for clustering

Supplemental Table 6: Cluster-based gene ontology pathways

Supplemental Table 7: GOMCL ontology network analysis

Supplemental Table 8: Differential DNA methylation analysis

Supplemental Table 9: Genomic feature distribution analysis

Supplemental Table 10: Over-represented KEGG pathways from differentially methylated CpG sites

Supplemental Table 11: Overlapping differential gene expression and DNA methylation changes

Supplemental Table 12: Overlapping DEGs and differentially methylated CpG sites KEGG pathway enrichment

Supplemental Figure 1: qPCR validation of RNAseq differentially expressed genes.

Supplemental Figure 2: KEGG pathways of differentially methylated CpGs

Supplemental Figure 3: Correlations between gene expression and DNA methylation changes
